# TBDB: a database of structurally annotated T-box riboswitch:tRNA pairs

**DOI:** 10.1093/nar/gkaa721

**Published:** 2020-09-03

**Authors:** Jorge A Marchand, Merrick D Pierson Smela, Thomas H H Jordan, Kamesh Narasimhan, George M Church

**Affiliations:** Department of Genetics, Harvard Medical School, Boston, MA 02115, USA; Department of Genetics, Harvard Medical School, Boston, MA 02115, USA; Department of Chemistry and Chemical Biology, Harvard University, Cambridge, MA 02138, USA; Wyss Institute for Biologically Inspired Engineering, Boston, MA 02115, USA; Institute of Chemical Sciences and Engineering, École Polytechnique Fédérale de Lausanne (EPFL), Lausanne, Switzerland; Department of Genetics, Harvard Medical School, Boston, MA 02115, USA; Department of Genetics, Harvard Medical School, Boston, MA 02115, USA; Wyss Institute for Biologically Inspired Engineering, Boston, MA 02115, USA

## Abstract

T-box riboswitches constitute a large family of tRNA-binding leader sequences that play a central role in gene regulation in many gram-positive bacteria. Accurate inference of the tRNA binding to T-box riboswitches is critical to predict their cis-regulatory activity. However, there is no central repository of information on the tRNA binding specificities of T-box riboswitches, and *de novo* prediction of binding specificities requires advanced knowledge of computational tools to annotate riboswitch secondary structure features. Here, we present the T-box Riboswitch Annotation Database (TBDB, https://tbdb.io), an open-access database with a collection of 23,535 T-box riboswitch sequences, spanning the major phyla of 3,632 bacterial species. Among structural predictions, the TBDB also identifies specifier sequences, cognate tRNA binding partners, and downstream regulatory targets. To our knowledge, the TBDB presents the largest collection of feature, sequence, and structural annotations carried out on this important family of regulatory RNA.

## INTRODUCTION

Bacteria exploit a wide-range of cis-acting RNA regulatory elements to control gene expression in response to specific environmental stimuli. One strategy used for modulating gene expression involves using 5′-UTR leader riboswitches to regulate transcription or translation (1–3). The transcriptional or translational logic of riboswitch leader sequences are conditionally dependent on the binding of a specific ligand (4).

In the gram-positive model organism *Bacillus subtilis*, an analysis of cis-regulatory sequences in the upstream region of several aminoacyl-tRNA synthetase (ARS) genes revealed that non-aminoacylated-tRNAs can act as a positive regulator (5,6). The discovery of this regulatory mechanism was a major breakthrough in understanding the expression of ARSs under nutrient limiting conditions. The T-box leader sequence was the first classical ‘riboswitch’ family to be discovered, preceding the discovering of metabolite-binding ribo-regulators (6).

**Figure 1.**
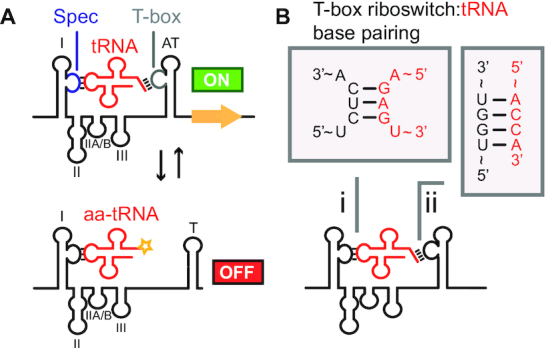
T-box riboswitches are cis-regulatory elements that use tRNA as a ligand. T-box riboswitches control translation or transcription of downstream genes. When uncharged cognate tRNA binds the T-box riboswitch, transcription or translation can proceed through stabilization of the antiterminator/antisequestrator structures. If charged cognate tRNA binds the T-box riboswitch, a terminator/sequestrator secondary structure forms preventing transcription or translation of downstream gene. (**A**) An archetypal ‘two-state’ conformational switch of a transcriptional T-box riboswitch is shown with structural features labeled: I = Stem I; II = Stem II; IIA/B = Stem IIA/B; III = Stem III; AT = antiterminator; T = terminator; Spec = specifier sequence (blue); T-box = T-box 5′-UGGN-3′ sequence (gray). (**B**) Watson–Crick base pairing between T-box riboswitch and tRNA in two critical regions dictate T-box riboswitch binding specificity: (i) specifier:anticodon base pairing dictates tRNA specificity while (ii) T-box bulge 5′-UGGN-3′ sequence:tRNA acceptor end base pairing controls regulatory logic.

T-box riboswitch leader sequences can link either transcription or translation of downstream genes to the aminoacylation-state of tRNA (7–10). In transcriptional regulation, the 3′-end of T-box leader sequences folds into either a terminator structure, prematurely stopping transcription, or antiterminator structure, allowing transcription to proceed (Figure 1A). Translational regulation occurs through a similar two-state mechanism, whereby a ribosome-binding site is either structurally sequestered, preventing ribosome binding or exposed, allowing ribosome binding and translation (8).

Mechanistic studies have revealed several interactions between T-box riboswitches and tRNAs. Classical Watson–Crick base pairing has been shown to occur between the T-box riboswitch specifier sequence and the tRNA anticodon (11). Additional Watson-Crick base pairing between the tRNA acceptor end (5′-NCCA-3′) and the first four residues of the T-box bulge (5′-UGGN-3′) has also been shown, and is thought to be the source of control for regulatory logic (Figure 1B) (12,13). If uncharged tRNA binds to the T-box riboswitch, Watson–Crick base pairing between the T-box bulge 5′-UGGN-3′ sequence and tRNA acceptor end, along with additional stacking interactions, results in stabilization of the antiterminator (for transcriptional) or antisequestrator (for translational) folds. Additional contacts between the antiterminator:tRNA heteroduplex minor groove and conserved purines in Stem III stabilize this interaction (14,15). If instead charged-tRNA binds the T-box bulge 5′-UGGN-3′, steric clashes prevent the full antiterminator/antisequestrator complex from forming leading the T-box riboswitch to adopt a terminator fold (for transcriptional regulation) or sequestrator fold (for translational regulation) (10,15).

The intricate and specific interactions between the T-box riboswitch:tRNA pair can be leveraged for a variety of applications across basic research and bioengineering. For example, a recent study used a *glyQS* T-box riboswitch to engineer a ribozyme that can specifically charge tRNA^Gly^ for use in cell-free protein synthesis (16). T-box riboswitches also have the potential to be used as a generalizable ‘registry-of-parts’, capable of independently sensing amino acid levels in the environment (17). Furthermore, due to their prevalence and importance in gram-positive bacteria, T-box riboswitches are also being studied as targets for antibiotics (18,19). Bacterial genomes tend to have several uncharacterized genes with remote homologs whose functions cannot be reliably predicted from sequence similarity alone. In this regard, T-box riboswitch specifier prediction has been used as a tool to uncover the function of unknown cis-regulated genes (20,21). In one case, the predicted T-box riboswitch family was used to infer the substrate specificity of downstream amino acid transporters (22).

Despite the identification of several thousand leader sequences across various databases, T-box riboswitch structures and functions remain under-characterized (20–28). Structural features of T-box riboswitches are critical to function and provide information regarding the evolution of lineage-specific T-box riboswitch sequences and respective structural adaptations (Supplementary Figure S1). For instance, the Stem I region varies widely in length, with the transcriptional T-box riboswitches having Stem I that are longer than those found in translational T-box riboswitches (8,20,21,29). Previous phylogenetic studies have also uncovered T-box riboswitch duplication events as well as changes in anticodon specificity in the Stem I region, suggesting a complex evolutionary history (21). Structural studies have also highlighted important sequence motifs in Stem II, such as the presence of a 5-purine string in the conserved S-turn that monitors the geometry of the specifier:anticodon interaction and even an F-box sequence in the Stem IIA/B region involved in pseudo-knot formation (30). Stem III has also been recognized as an integral part of the aminoacylation sensing domain. Sequence analysis reveals the presence of a conserved 5′-RRRNG-Stem III-AA-3′ motif responsible for rejection of 2′-aminoacylated tRNAs (15). However, existing public databases which host putative T-box leader sequences do not attempt to fold and annotate structural features nor include potential tRNA binding specificities for T-box riboswitches. Currently, *in silico* structure prediction and feature extraction is required to both predict conserved structural regions and substrates from raw sequences, and therefore exists as a barrier for entry to anyone interested in T-box riboswitch research.

**Figure 2.**
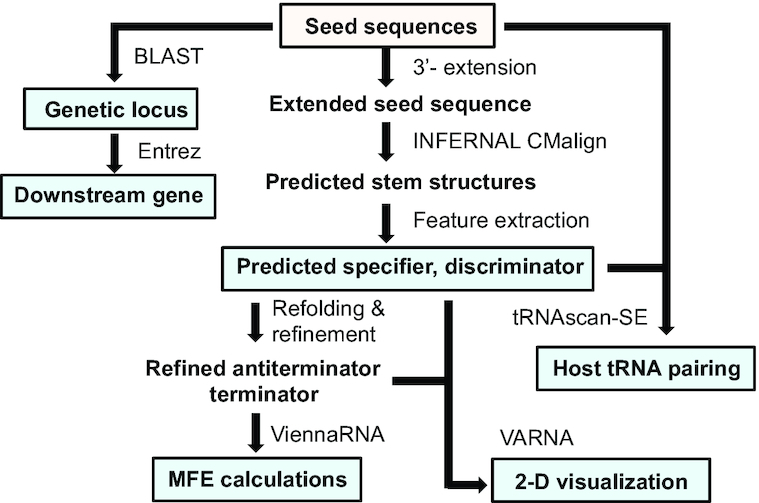
Construction of TBDB. T-box riboswitch structures were predicted from input sequences using INFERNAL and RNAfold (Vienna RNA) (35,36). T-box riboswitch features (specifier sequence and T-box riboswitch sequence) were extracted from structural predictions. For input sequences where genomic information was not provided, BLAST (NCBI) was used to identify genetic locus within host. Entrez (NCBI) queries were used to compile all genomic sequence records of the host organisms including genes found downstream of T-box riboswitch input sequences. tRNAscan-SE was run on all genomes to find tRNAs in the hosts with anticodons that are reverse complements of T-box riboswitch specifier sequences (39). Predicted structures were refined using ViennaRNA (36). Refined structures, with predicted features, were visualized as 2D representations using VARNA (40). Minimum free energy (MFE) calculations were performed using ViennaRNA on refined terminator and antiterminator/antisequestrator structures.

Here, we present the T-box Riboswitch Annotation Database (TBDB), a compilation of T-box riboswitch sequences from various primary sources with detailed annotations to aid future research. The TBDB predicts putative transcriptional and translational T-box riboswitch sequences, annotates secondary structures, identifies functional features and downstream genes, finds cognate pairs of tRNAs from host organisms, calculates MFE (minimum free-energy) structures and provides rich visualization for known and predicted T-box riboswitch leader sequences (Figure 2). The TBDB is browsable at https://tbdb.io, with the entire database available to download as a single flat file. The TBDB aims to be a valuable resource for studying canonical, engineered, and mutant T-box riboswitch mechanisms and will provide a point-of-entry for studying regulation and interactions between T-box riboswitch:tRNA pairs (Supplementary Table 1). As a resource for the wider non-coding RNA community, the TBDB is the first structural and functionally annotated database for studying gene regulation by T-box riboswitches.

## MATERIALS AND METHODS

### Data collection and sequence curation

Class I T-box riboswitch (transcriptional) leader sequences used to generate the database were obtained from a variety of primary sources including the RFAM 14.0 database, RibEX database, GeConT3 database and others (21,31–34). For class I transcriptional T-box riboswitch leader sequences that did not necessarily contain the terminator (RFAM and GeConT3 sequences) Entrez (NCBI) was used to extend sequences by 50 nt. Extended sequences were then subsequently trimmed to end in penta poly-U (3′-UUUUU). Sequences from these databases that were too short (<100 nt), too long (>500 nt), or redundant were also removed. Sequences of translational T-box riboswitches (class II) were found by using our custom covariance model (Supplementary Figure S2) to perform an INFERNAL search on all NCBI reference genomes in the Actinobacteria phylum (TaxID:1760) (35). The class II covariance model was additionally applied on T-box riboswitch sequences detected by the class I model but predicted to have a truncated Stem I (Supplementary Figure S3).

### Structural and thermodynamic prediction of T-box riboswitch leaders

INFERNAL was used to predict the secondary structure of input sequences using either the RFAM 14.0 class I T-box riboswitch covariance model (RF00230.cm) (34,35), or our class II T-box riboswitch covariance model (Supplementary Figure S4). The INFERNAL output structure, corresponding to the antiterminator/antisequestrator fold, was then used to predict T-box riboswitch features according to the conserved patterns of stems and loops. Next, the MFE (Supplementary Figure S5) of the structure was evaluated using RNAfold (ViennaRNA) (36). For putative transcriptional T-box riboswitches, the terminator structure was determined by searching for a suitable terminator hairpin using RNALfold. The MFEs of both folds were calculated using RNAeval. Structures for sequestrator stems from translational T-box riboswitches are not currently predicted. Summary of MFE for T-box riboswitches represented in the TBDB are shown in Supplementary Table 2. Detailed descriptions of the feature extraction and thermodynamic prediction are available in the Supporting Information.

### Pairing T-box riboswitches with putative cognate tRNAs

Top specifier sequence predictions were used to identify a putative tRNA family pair for each T-box riboswitch. To find the sequence of cognate tRNA, Entrez (NCBI) was used to query for all genomic records of the T-box riboswitch host organism. Genome sequences, either partial or full, were downloaded from RefSeq or GenBank (37,38). tRNAscan-SE (Lowe Lab, UCSC) was used to identify tRNAs in each host organism (39). tRNAs with predicted anticodons matching the specifier were considered paired. For cases where more than one possible tRNA gene was possible, a single tRNA was chosen from among the matching tRNAs for display. tRNA visualization was generated using VARNA (40).

### Prediction of T-box riboswitch specifier sequence

The T-box riboswitch specifier region was assigned as the 1-5 bp (inclusive) 5′- from the end of the Stem I specifier bulge. In these five bases, three possible specifier sequence frames (‘−1’, ‘+0’, ‘+1’) were examined for meeting specifier-match criteria. For each possible specifier, we identified the putative tRNA family (by matching anticodon) that would bind. We then checked to see if (i) the predicted tRNA family had a discriminator base that could base pair with the T-box 5′-UGGN-3′ sequence, with wobble allowed, and (ii) if the predicted tRNA amino acid family matched the downstream gene ontology (where available). In the case of predicted His T-box riboswitches, discriminator matching was not used as a criterion as mature tRNA^His^ transcripts can have a paired discriminator base (41).

For matching T-box 5′-UGGN-3′ with tRNA acceptor end 5′-NCCA-3′ sequence, we first searched the host for all tRNAs of a given tRNA family and identified which discriminator base that specific host used for a particular tRNA. In cases where we could not identify matching tRNAs in the host organism, the bacterial discriminator base frequency information was extracted from tRNAviz and utilized in the specifier prediction model (42). The top specifier was then assigned as the specifier that met most of these conditions, equally weighted, with preference given in the following order: ‘+0’ > ‘−1’ > ‘+1’ specifier sequence frames. In cases where more than one specifier was possible, the top specifier was assigned as mentioned but alternative specifier sequence frames are also provided in TBDB.

## RESULTS AND DISCUSSION

### Accessing TBDB content

The TBDB aims to be a comprehensive and approachable hub for predictions of T-box riboswitch structure and function. Database entries are provided in a searchable, tabulated format. Users can query entries based on fields that include sequence, host organism, specifier sequence, T-box 5′-UGGN-3′ sequence, or predicted tRNA family (Supplementary Figure S6).

Detailed information on each TBDB entry can be obtained by accessing the unique ID in the database table. Doing so brings users to a T-box riboswitch entry page that contains source, downstream protein annotation, structural, functional, and sequence information. The title of the entry page provides the T-box riboswitch ID, a unique identifier generated by TBDB, as well as the predicted tRNA family the T-box riboswitch interacts with in the host organism (Supplementary Figure S7). The source information panel gives a high-level summary of the T-box riboswitch entry and includes information regarding genetic locus and feature predictions. The following panel provides an interactive genome browser (NCBI) starting at the T-box leader sequence locus and ending 5,000 bp downstream. The genome browser allows users to visualize the genomic context of T-box riboswitch leader sequences and provides a quick method to assess the validity of T-box riboswitch specifier predictions. For example, a Trp T-box riboswitch is observed as a 5′-UTR of an operon involved in tryptophan biosynthesis (Supplementary Figure S8).

Towards the goal of making a T-box riboswitch registry-of-parts, in the subsequent panel we provide a T-box riboswitch sequence that stretches from the Stem I to the terminator poly-U region (for transcriptional T-box riboswitches, Supplementary Figure S9). Visualizations (VARNA) of the predicted secondary structures of the T-box riboswitch are given in the next panel (40). These 2D representations highlight important features of the T-box riboswitch entry including Stem I (light-yellow), possible specifier sequence (dark-yellow), antiterminator/antisequestrator (light-blue), the four nucleotide 5′-UGGN-3′ in the T-box bulge (dark blue), and terminator stem (red). The dot-bracket representations of the 2D structures are also provided (Supplementary Figure S10). Results for tRNA matching, generated using tRNAscan-SE, can be found in the following panel (Supplementary Figure S11) (39). Here, we show the sequence and description for the highest scoring predicted tRNA, with matching anticodon, in T-box riboswitch host organism (if available). tRNAs for alternative specifier sequence frames are also generated if our model could not identify a consensus specifier. Minimum free energy (MFE) predictions for each of the folds are given in the thermodynamics section, and are the result of structure refinement performed using ViennaRNA (Supplementary Figure S12) (36).

Finally, the INFERNAL panel has output information from structural searches, which can be used as a reference by users interested in structure prediction information and quality (Supplementary Figure S13). Here, users will also find information about predicted boundaries of important structural features. We believe the TBDB sequence demarcation features of the important Stem I, Stem II, Stem III, antiterminator/antisequestrator and terminator/sequestrator regions will spur future efforts to explore common themes and diversity that T-box riboswitches have accrued and help aid in their classification.

### Identification of T-box riboswitch:tRNA pairs

T-box riboswitches tend to have a strict preference for canonical Watson–Crick base pairing between the specifier sequence and the anticodon of the cognate tRNA (9). Depending on the length of the specifier bulge, it is also likely that there are alternative specifier sequences, allowing for the possibility of multi-tRNA specificity in gene regulation (43). However, experimental work uncovering the determinants of multi-specificity in T-box riboswitches remains sparse. Our specifier prediction assignment takes into consideration downstream gene ontology and base pairing between the T-box bulge 5′-UGGN-3′ sequence and the tRNA acceptor end found in the host organism. In assigning T-box riboswitch specifier, priority was given to as the region 2-4 bp (inclusive) 5′- from the end of the Stem I specifier bulge, though ‘–1’ and ‘+1’ specifier sequence frames were also considered (Supplementary Figure S1). In a majority of the cases, the variable position (5′-UGGN-3′) on the T-box bulge shows Watson-Crick base pairing with the discriminator base (5′-NCCA-3′) of the cognate tRNA species. Exceptions were noted in the Trp family, where 46% of T-box riboswitches in our collection have a ‘U’ at the degenerate position while their cognate tRNAs have a ‘G’ at the discriminator position, suggesting a G:U wobble pair, as has been previously noted (41). Based on this observation we allowed for wobble base pairs between the tRNA discriminator and the degenerate nucleotide in the 5′-UGGN-3′ region of the T-box bulge sequence in our final model. In the case of putative His T-box riboswitches, discriminator base matching was not considered as tRNA^His^ transcripts can have an internally paired discriminator base (41).

In practice, the TBDB identifies the cognate tRNA pairs from T-box riboswitch hosts by first predicting the specifier sequence, then searching genome records of respective hosts for tRNAs that have a matching anticodon (Watson–Crick base pairing, no wobble allowed) and discriminator base pairing (both Watson-Crick and wobble pairing allowed). Our model gave a single specifier sequence frame prediction for 16,258 T-box riboswitch leader sequences, two possible specifier sequence frames for 2,884 sequences, and three specifier sequence frames for 3,551 sequences. For 48 sequences, we were able to predict a specifier but were unable to find the canonical 5′-UGGN-3′ sequence in the T-box bulge. In cases where more than one specifier is possible, preference is given to the ‘+0’ specifier sequence frame (Supplementary Figure S1C). In all, T-box riboswitch leaders containing Trp-, Leu- and Ile- tRNA matching specifiers were most commonly observed in our collection while Lys-, Glu- and Gln- matching specifiers were the least common, well in agreement with previous findings (20). Supplementary Table 1A and B show composition of the T-box Riboswitch Annotation Database amino acid family and specifier usage.

Through our tRNA search, we were able to match 79.4% of T-box riboswitches for which we predicted a specifier with a tRNA of the native host. Grouping T-box riboswitches by specifier sequence, we found that tRNA-matching was >80% for most specifiers (Supplementary Table 1B). Interestingly, we only identified a tRNA pair for 8.6% of T-box riboswitches with 3′-U specifiers, consistent with observations that 5′-A starting anticodons in bacterial tRNAs are rare (Supplementary Table 1C). In these cases, it is possible that these T-box riboswitches are controlled by tRNAs without matching anticodons (e.g. relying on wobble base pairing) or are using an alternative specifier sequence frame when binding anticodons (44). Alternative specifier sequence frames for T-box riboswitch sequences have previously been observed in at least one experimentally studied system (43).

### T-box riboswitch specifier usage

Identifying specifier sequences for T-box riboswitches allowed us to interrogate the choice of tRNA anticodon, and therefore the tRNA, that is used for regulation. Analogous to ‘codon usage tables’, which summarize an organism’s codon preference for translating particular amino acid, Table 1 depicts a ‘specifier usage table’ generated from 23,535 sequences in the TBDB. For T-box riboswitches of amino acid families that only have two codons (Lys, Asp, Asn, Glu, Gln, His, Tyr, Cys, Phe), a single specifier is preferred in over 85% of corresponding T-box riboswitch sequences. The choice of specifier is also consistent, with 3′-A and 3′-C always favored over 3′-G and 3′-U respectively. T-box riboswitches of amino acid families decoded by four codons (Gly, Ala, Pro, Val, Thr) have more diversity in specifier usage. Much like the two-codon sets, there is a preference for Val, Thr and Gly T-box riboswitches to use 3′-A and 3′-C specifiers. Interestingly, Ala and Pro T-box riboswitches display a preference for 3′-U specifiers. T-box riboswitch families for amino acids with 6 codons (Leu, Ser, Arg) show a 3′-A and 3′-C preference, with an even stronger preference for 3′-C specifiers. In the particular case of Leu family T-box riboswitches, the CUC specifier is observed in 74% of sequences. For the special case of Ile, 3′-C (AUC) specifier is preferred. The large collection of sequences has allowed us to reinforce previous observations that the ‘C-rule’ (3′-C in specifiers) is prevalent, while additionally discovering that 3′-A usage is also largely preferred for specific amino acid classes (Supplementary Table 1B and C) (20,21,32). Indeed, recent structural analysis reveals that a non-canonical A-minor motif in the Stem II S-turn places local constraints directly to prefer a guanine on the tRNA anticodon, and therefore a cytosine in the third position of the specifier (15,30).

**Table 1.** Specifier usage table based on ‘Top’ specifier predictions for T-box riboswitch leader sequences in the TBDB. Frequency given on an amino acid basis for the entire sequence collection in the TBDB. Specifier usage frequencies were rounded to two decimal places.

SPEC	AA	FREQ	SPEC	AA	FREQ	SPEC	AA	FREQ	SPEC	AA	FREQ
AAA	K	0.87	AGA	R	0.24	ACA	T	0.13	AUA	I	0.01
AAG	K	0.13	AGG	R	0.04	ACG	T	0.01	AUG	M	1.00
AAC	N	0.98	AGC	S	0.09	ACC	T	0.60	AUC	I	0.99
AAU	N	0.02	AGU	S	0.01	ACU	T	0.25	AUU	I	0.01
GAA	E	0.88	GGA	G	0.17	GCA	A	0.18	GUA	V	0.61
GAG	E	0.12	GGG	G	0.04	GCG	A	0.01	GUG	V	0.02
GAC	D	0.94	GGC	G	0.77	GCC	A	0.09	GUC	V	0.29
GAU	D	0.06	GGU	G	0.02	GCU	A	0.72	GUU	V	0.08
CAA	Q	0.95	CGA	R	0.01	CCA	P	0.16	CUA	L	0.02
CAG	Q	0.05	CGG	R	0.08	CCG	P	0.10	CUG	L	0.07
CAC	H	0.95	CGC	R	0.52	CCC	P	0.14	CUC	L	0.77
CAU	H	0.05	CGU	R	0.12	CCU	P	0.60	CUU	L	0.11
UAA	*	0.00	UGA	*	1.00	UCA	S	0.06	UUA	L	0.01
UAG	*	0.00	UGG	W	1.00	UCG	S	0.03	UUG	L	0.02
UAC	Y	0.99	UGC	C	1.00	UCC	S	0.65	UUC	F	0.99
UAU	Y	0.01	UGU	C	0.00	UCU	S	0.16	UUU	F	0.01

There are possible explanations for the source of specifier-usage bias in T-box riboswitches. First, specifier usage does not follow the same observed patterns of codon usage. In most cases, the preferred specifier is the least preferred codon for the amino acid family. For example in the taxonomic order Bacillales, the Phe UUU codon is used in approximately 70% of cases for translation (45), but is present in only 1% of Phe T-box riboswitch specifiers. One possible hypothesis for the specifier-use bias could be attributed to T-box riboswitches favoring interaction with a single tRNA species and disfavoring wobble base pairing. In the absence of tRNA with 5′-I (Inosine) anticodons, 3′-A and 3′-C codons are only decoded by a single tRNA species (5′-U and 5′-C anticodons), whereas 3′-G and 3′-U codons can be decoded by multiple tRNAs (5′-U/C and 5′-A/G anticodons). T-box riboswitches likely co-evolved to be highly specific in their response towards a single tRNA species, which would have been made more difficult if specifier binding is made competitive with two (or more) tRNA species (Watson-Crick basepair versus wobble). Additionally, tRNAs with 5′-A anticodons are not prevalent in bacteria, as the U:G wobble-pair is the preferred mechanism for decoding 3′-U codons (46). The consensus sequence of the 23,535 T-box riboswitches (Supplementary Figure S1) revealed that 5′-NNC-3′ specifiers were preferred overall, being represented at 59.9% of T-box riboswitch sequences with predicted specifiers.

### Tool for T-box riboswitch scanning and feature extraction

In order to increase accessibility and reproducibility for detection and annotation of T-box riboswitches for arbitrary DNA sequences, we have also released a standalone CLI tool (tbox-scan) for finding and extracting features of T-box riboswitches. Tbox-scan uses INFERNAL to find T-box riboswitches in a FASTA sequence input, and then performs the same feature extraction used to build the TBDB. As outputs, the tool displays T-box riboswitch location, specifier sequence, T-box bulge 5′-UGGN-3′ sequence, and secondary structure prediction of antiterminator/antisequestrator and terminator folds. Covariance models for putative transcriptional (class I) and putative translational (class II) T-box riboswitches are also provided. Users can download tbox-scan from https://tbdb.io/tools/tbox-scan.html.

## CONCLUSION

T-box leader sequences were the first riboswitches to be discovered, yet remain under characterized. Of the known >20,000 sequences, few have been tested for regulatory activity. Currently, T-box riboswitch research is stymied by the necessity of secondary structure modeling to resolve which tRNA binds a given T-box riboswitch. Through a compilation of sequence information from multiple sources, the TBDB increases access to T-box riboswitch functional information. The TBDB has aggregated and processed over 23,000 T-box riboswitch sequences from 3,632 bacterial species in order to identify structural features and tRNA binding partners (Supplementary Figure S14). The TBDB aims to be an approachable hub for the riboswitch community and in future version aims to integrate well with experiments for both natural and engineered T-box riboswitches. As more experiments are being carried out in this area, additional feature annotations will become available - such as predictive models for tandem T-box riboswitches, structural annotation of Stem IIA/B pseudoknots, K-turn motifs, sub-structure analysis, and mutability of synthetic T-box riboswitches.

## DATA AVAILABILITY

The TBDB is free to access and does not require user registration to use. The database and tools are accessible to browse at https://tbdb.io. All data used to generate TBDB can be accessed for download at https://tbdb.io/download/tbdb.csv. Documentation and package for the tbox-scan tool are available at https://tbdb.io/tools/tbox-scan.html. The full pipeline used to generate entries in TBDB (from FASTA to TBDB entry) is available to download in our repository (https://github.com/mpiersonsmela/tbox/).

## Supplementary Material

gkaa721_Supplemental_Files
