# Supplementary material for: TBDB: a database of structurally annotated T-box riboswitch:tRNA pairs
Source: Nucleic Acids Res. 2020 Sep 3;49(D1):D229–35. doi: 10.1093/nar/gkaa721 (PMC7778990; doi:10.1093/nar/gkaa721)

Supplemental Information: TBDB – A database of structurally annotated T-box riboswitch:tRNA pairs

Jorge A. Marchand[1,‡], Merrick D. Pierson Smela[1,2,‡], Thomas H. H. Jordan[3], Kamesh Narasimhan[1,*], George M. Church [1,4,*]

[1]Department of Genetics, Harvard Medical School, Boston, MA 02115
[2]Department of Chemistry and Chemical Biology, Harvard University, Cambridge, MA 02138
[3]Institute of Chemical Sciences and Engineering, Ecole Polytechnique Fédérale de Lausanne (EPFL), Lausanne, Switzerland
[4]Wyss Institute for Biologically Inspired Engineering, Boston, MA, 02115 USA
‡The authors wish it to be known that, in their opinion, the first two authors should be regarded as joint First Authors.
*To whom correspondence should be addressed. Email: kamesh_narasimhan@hms.harvard.edu Correspondence may also be addressed to gchurch@genetics.med.harvard.edu

Supplementary Discussion

Sourcing and curation of the TBDB S2
Distribution of T-box riboswitches in TBDB by phyla S2

Supplementary Methods

Covariance model generation for Class II isoleucyl translational T-box riboswitches S4
Feature extraction algorithm S4
T-box riboswitch structure refinement. S4
Thermodynamic calculations S4
Identification of downstream gene S5
Benchmarking specifier feature prediction S5
Visualization of T-box riboswitch structures. S5

Supplementary Tables and Figures

Table S1. *Sequence composition of the T-box riboswitch Database* S7
Table S2. *Summary of RNA folding energies for sequences in the database* S9
Fig S1. *Consensus sequence and structure for T-box riboswitch and tRNA families in the TBDB* S10
Fig S2. *R2R representation of Class II T-box riboswitch model generated* S11
Fig S3. *Stem I lengths in TBDB* S12
Fig S4. *INFERNAL score density plot for sequences in the TBDB* S13
Fig S5. *Distribution of minimum free energy structures in the TBDB* S14
Fig S6. *The TBDB interface – Database table* S15
Fig S7. *The TBDB interface – Entry title and source information* S16
Fig S8. *The TBDB interface – Genomic context viewer* S17
Fig S9. *The TBDB interface – T-box riboswitch FASTA sequence* S18
Fig S10. *The TBDB interface – Predicted secondary structure visualization* S19
Fig S11. *The TBDB interface – tRNA scan results* S20
Fig S12 *The TBDB interface – MFE predictions* S21
Fig S13. *The TBDB interface – INFERNAL prediction output* S22
Fig S14. *Composition of TBDB by phyla and T-box riboswitch family.* S23

SUPPLEMENTAL TEXT

Sourcing and curation of the TBDB.

The T-box riboswitch annotation Database comprises 23,535 non-redundant T-box riboswitch sequences. Sequences used to build the TBDB were sourced from work that either used covariance models or motif analysis for T-box riboswitch sequence discovery [1–6]. Many of the input sequences used to generate the TBDB were shared across the various databases with varying definitions of T-box riboswitch start and end. We used an INFERNAL covariance model (RFAM RF00230) to predict class I transcriptional T-box riboswitch structure from T-box riboswitch input sequences [6,7]. For class II translational T-box riboswitches, a custom covariance model was generated using seed sequences for class II translational T-box riboswitches found in previously published work [4,8]. A representation of the class II translational T-box riboswitch model is shown in **Figure S2**, and was generated using R2R [9]. Representation for Class I T-box riboswitch model can be found on RFAM (http://rfam.xfam.org/family/RF00230). The INFERNAL log-odds score distribution of the predicted T-box riboswitches is shown in **Figure S4**. T-box riboswitch structures were subsequently hashed to generate a unique identifier, forming the basis for the TBDB unique ID. The distribution of Stem I structure lengths in the TBDB is shown in **Figure S3**.

In order to further curate the database, we only made tRNA predictions for sequences with predicted structures that properly fold into canonical Stem I and antiterminator regions. Proper *in silico* folding of these two regions are critical for identification of specifier sequence and T-box riboswitch sequence. Class I T-box riboswitch sequences for which we could not predict a plausible terminator stem are kept in the database, as they could be members of class I translational control T-box riboswitches, which do not contain terminators but rather sequestrator stems. In the interest of preserving sequence information, T-box riboswitches with no structural predictions are displayed in TBDB though only genetic context is available as additional information. For transcriptional T-box riboswitches, *in silico* folding of T-box riboswitch terminator and antiterminator regions were used as an additional metric to examine plausibility of structure predictions (**Figure S5**). The free-energy values for the folds predicted in the antiterminator and terminator regions (ΔG_{anti}= -7.4 ± 4.2 kcal/mol, ΔG_{term} = -19.5 ± 7.9 kcal/mol) is largely consistent with the expectation that terminator hairpin is more stable than the antiterminator. In all, the TBDB contains 20,342 putative transcriptional T-box riboswitches, 1,014 putative translational T-box riboswitches, and 2,179 of unknown regulation.

Distribution of T-box riboswitches in TBDB by phyla

A majority of the transcriptional T-box riboswitch collection in TBDB primarily derives from gram-positive phylum Firmicutes (**Figure S14**) [10]. In particular, sequences in the Bacilli and Clostridia family, within Firmicutes, constitute the majority of T-box riboswitch sequences. T-box riboswitches with

specifiers matching all the standard 20 tRNA families are observed in Bacilli and Clostridia, and agrees with the prevailing notion that these bacteria use T-box riboswitches as primary regulators for controlling aminoacylation homeostasis. For non-Firmicutes sequences in the TBDB, the diversity and distribution of T-box riboswitch does not follow the trend within Firmicutes and likely reflect phyla specific usage and adaptation. The other major family represented in the TBDB collection are the Ile translational T-box riboswitches derived from Actinobacteria. Overall the Ile, Trp, Leu, Met and Val families constitute 50% of the T-box riboswitches in TBDB. The diversity of T-box riboswitches in Firmicutes, and relative lack thereof in other phyla, brings to question their origins. The distribution of T-box riboswitches in TBDB conforms to previous observations that T-box riboswitches likely evolved in a common ancestor of Firmicutes and the Actinobacteria, the Chloroflexi, and the Deinococcus-Thermus (DT) group while being distributed across other phyla through horizontal gene transfer (HGT) events [11].

SUPPLEMENTAL METHODS

Covariance model generation for Class II isoleucyl translational T-box riboswitches. A set of 115 unique *ileS* leader sequences was generated by BLAST search using 37 published *ileS* T-box riboswitch sequences as inputs [4,8]. These sequences were aligned using mlocarna, and the multiple sequence alignment was manually annotated with secondary structure corresponding to the published structure of the *Nocardia farcinica ileS* T-box riboswitch [12,13]. Next, INFERNAL was used to construct a covariance model while iteratively refining the alignment. The covariance model used to identify class II translational Ile T-box riboswitches is available in our repository and is visualized in **Figure S2**.

Feature extraction algorithm. The first stem-loop in the secondary structure was assigned as Stem I. For Class I transcriptional T-box riboswitches, the last bulge within the Stem 1 structure was assigned as the specifier bulge. For Class II translational T-box riboswitches, the specifier loop was assigned as the Stem 1 hairpin loop. The specifier sequence was assigned as the bases 2-4 (inclusive) before the 3′-end of the specifier bulge (**Figure S1C**). The antiterminator was assigned as the last stem-loop predicted by the covariance model. The T-box UGGN sequence was assigned as the first unpaired 5′-UGGN-3′ sequence within the T-box antiterminator bulge, and the antidiscriminator base was assigned as the last base of this sequence.

T-box riboswitch structure refinement. The INFERNAL predicted secondary structure and sequence were processed to remove gaps and truncations. For each entry, truncations were filled from the corresponding FASTA sequence. Gaps in the aligned sequence and their corresponding structural characters in the dot-bracket notation were removed. Pairs between nucleotides and gaps in the INFERNAL predicted structure were unpaired by removing the gap/bracket from the sequence/structure, respectively, and changing the paired bracket to a dot. Energetically unfavorable hairpin loops of 0, 1, or 2 nt were expanded into loops of 3 or greater. Antiterminator structures were further refined by 1) using the INFERNAL antiterminator structure as a soft constraint for RNAfold (ViennaRNA) and 2) using the discriminator and the nucleotides downstream of the antiterminator unpaired using hard constraints [14].

The terminator region was found by searching for the first poly-U sequence (defined as a seven-nucleotide region containing at least five U's) starting 10 nt after the end of the antiterminator hairpin. Once this sequence was identified, RNALfold was used to generate a list of candidate terminator hairpins between the discriminator region and the poly-U region. Next, this list was filtered to remove hairpins with stem length shorter than 6 nt, as well as hairpins ending more than 2 nt before the poly-U region. The terminator hairpin was chosen as the largest hairpin among this filtered list, and the energy was evaluated using RNAeval. If this search method failed, due to lack of a poly-U region or otherwise, a fallback

method simply used RNAfold to find the minimum free energy structure of the sequence from the antiterminator start to the end of the T-box riboswitch. Energies of terminators predicted by the fallback method were not included in analysis. Terminator energies were not predicted for class II translational T-box riboswitches.

Thermodynamic calculations. RNAeval (ViennaRNA) was used to calculate Gibbs free energy (MFE) of predicted secondary structures for terminator and antiterminator sequences [14]. The sequence and associated secondary structures starting at the antiterminator and ending at the poly-U if present were used to calculate ΔG_{anti} (kcal/mol) and ΔG_{term} (kcal/mol). Calculations were performed at 37 °C with default settings, allowing GU pairing. The free energy change between terminator and antiterminator conformations for the T-box riboswitch $\Delta\Delta G_{term\text{-}anti}$ was taken as the difference between ΔG_{term} and ΔG_{anti}. Thermodynamic contributions from tRNA binding were not taken into consideration.

Identification of downstream gene. The T-box riboswitch NCBI accession was used to identify the genetic locus of the T-box riboswitch within its host organism. An Entrez query for features 500 nt downstream of the T-box riboswitch end was used to retrieve downstream gene/protein description, protein accession, and enzyme commission number if available. The protein accession was then used to obtain additional gene ontology information by querying the appropriate database (Uniprot, KEGG, ENA, or DDBJ).

Benchmarking specifier feature prediction. The feature-annotated T-box riboswitch leader dataset from Vitreschak *et al.* was used to benchmark the pipeline for structure and feature prediction [4]. Of the initial 698 sequences from the curated input dataset, INFERNAL was able to detect T-box riboswitch leaders in 694 sequences (99.5%). Of these, 621 (89.5%) scored high enough to match both specifier bulge and T-box riboswitch sequence predictions. Our structure prediction pipeline was able to accurately predict 589 of 621 specifier sequences (94.8%) from the Vitreschak *et al.* dataset [4]. In one additional sequence the appropriate specifier was found in the "-1" specifier sequence frame (0.2%) and in 9 other sequences the correct specifier was found "+1" specifier sequence frame (1.4%) (**Figure S1C**). 22 sequences (3.5%) had specifier predictions that did not match the validation dataset, and include cases where specifier predictions were off by 2 or more nt.

Visualization of T-box riboswitch structures. The MFE structure predictions of the refined terminator and antiterminator structures were combined with the INFERNAL prediction structure of the Stem I, Stem III, and other regions, if present [7]. VARNA was used to convert structures from dot-bracket notation to 2D flat image representation [15].

REFERENCES

1. Abreu-Goodger, C. & Merino, E. RibEx : a web server for locating riboswitches and other conserved bacterial regulatory elements. *Nucleic Acids Res.* **33,** 690–692 (2005).
2. Martinez-Guerrero, C. E., Ciria, R., Abreu-Goodger, C., Moreno-Hagelsieb, G. & Merino, E. GeConT 2 : gene context analysis for orthologous proteins, conserved domains and metabolic pathways. *Nucleic Acids Res.* **36,** 176–180 (2008).
3. Weinreb, C. *et al.* 3D RNA and functional interactions from evolutionary couplings. *Cell* **165,** 963–975 (2016).
4. Vitreschak, A. G., Mironov, A. A., Lyubetsky, V. A. & Gelfand, M. S. Comparative genomic analysis of T-box regulatory systems in bacteria. *Rna* **14,** 717–735 (2008).
5. Griffiths-Jones, S., Bateman, A., Marshall, M., Khanna, A. & Eddy, S. R. Rfam : an RNA family database. *Nucleic Acids Res.* **31,** 439–441 (2003).
6. Kalvari, I. *et al.* Rfam 13.0: Shifting to a genome-centric resource for non-coding RNA families. *Nucleic Acids Res.* **46,** 335–342 (2018).
7. Nawrocki, E. P. & Eddy, S. R. Infernal 1.1: 100-fold faster RNA homology searches. *Bioinformatics* **29,** 2933–2935 (2013).
8. Sherwood, A. V., Grundy, F. J. & Henkin, T. M. T box riboswitches in Actinobacteria: Translational regulation via novel tRNA interactions. *Proc. Natl. Acad. Sci. U. S. A.* **112,** 1113–1118 (2015).
9. Weinberg, Z. & Breaker, R. R. R2R - software to speed the depiction of aesthetic consensus RNA secondary structures. (2011).
10. Grigg, J. C. *et al.* T box RNA decodes both the information content and geometry of tRNA to affect gene expression. *Proc. Natl. Acad. Sci. U. S. A.* **110,** 7240–7245 (2013).
11. Gutierrez-Preciado, A., Henkin, T. M., Grundy, F. J., Yanofsky, C. & Merino, E. Biochemical features and functional implications of the RNA-based T-Box regulatory mechanism. *Microbiol. Mol. Biol. Rev.* **73,** 36–61 (2009).
12. Suddala, K. C. & Zhang, J. High-affinity recognition of specific tRNAs by an mRNA anticodon-binding groove. *Nat. Struct. Mol. Biol.* **26,** 1114–1122 (2019).
13. Will, S., Reiche, K., Hofacker, I. L., Stadler, P. F. & Backofen, R. Inferring noncoding RNA families and classes by means of genome-scale structure-based clustering. *Plos Comput. Biol.* **3,** 680–691 (2007).
14. Lorenz, R. *et al.* ViennaRNA package 2.0. *Algorithms Mol. Biol.* **6,** 1–14 (2011).
15. Darty, K., Denise, A. & Ponty, Y. VARNA : Interactive drawing and editing of the RNA secondary structure. *Bioinformatics* **25,** 1974–1975 (2009).
16. Capella-Gutiérrez, S., Silla-Martínez, J. M. & Gabaldón, T. trimAl : a tool for automated alignment trimming in large-scale phylogenetic analyses. *Bioinformatics* **25,** 1972–1973 (2009).
17. Barter, R. L., Yu, B., Barter, R. L. & Yu, B. Superheat : An R package for creating beautiful and extendable heatmaps for visualizing complex data. *J. Comput. Graph. Stat.* (2018). doi:10.1080/10618600.2018.1473780

SUPPLEMENTAL TABLES AND FIGURES

Table S1. Sequence composition of the T-box Riboswitch Annotation Database.

A) Database composition by tRNA family. tRNA family lists the amino acid abbreviation of corresponding tRNA (SUP = suppressor tRNA; NONE = no tRNA predicted). '# Sequences (w/tRNA)' shows the number of T-box riboswitch sequences for which a matching tRNA was found in the host organism.

tRNA family	# Sequences	# Sequences (w/ tRNA)
ALA	755	159
ARG	704	319
ASN	754	636
ASP	510	451
CYS	879	837
GLN	226	217
GLU	51	43
GLY	557	524
HIS	475	424
ILE	3451	2896
LEU	2096	1479
LYS	170	157
MET	1951	1783
SUP	9	0
PHE	1485	1323
PRO	837	314
SER	1228	899
THR	1364	963
TRP	2344	2140
TYR	1460	1332
VAL	1635	1324
NONE	594	0

B) Database composition by predicted specifier sequence. 'SPEC' column indicates predicted specifier sequence (5′-3′), 'AA' column contains the abbreviation for the amino acid encoded by the anticodon that matches the specifier, 'SEQ' shows total number of sequences with predicted specifier in the TBDB, and 'w/ tRNA' shows the number of sequences where tRNAscan-SE found a matching tRNA in the host genome.

SPEC	AA	SEQ	w/tRNA	SPEC	AA	SEQ	w/tRNA	SPEC	AA	SEQ	w/tRNA	SPEC	AA	SEQ	w/tRNA
AAA	K	148	137	AGA	R	167	154	ACA	T	124	114	AUA	I	17	0
AAG	K	22	20	AGG	R	27	24	ACG	T	16	4	AUG	M	1951	1783
AAC	N	740	636	AGC	S	105	100	ACC	T	866	800	AUC	I	3417	2896
AAU	N	14	0	AGU	S	14	0	ACU	T	358	45	AUU	I	17	0
GAA	E	45	42	GGA	G	95	93	GCA	A	137	92	GUA	V	1002	873
GAG	E	6	1	GGG	G	20	10	GCG	A	4	1	GUG	V	33	12
GAC	D	477	451	GGC	G	431	421	GCC	A	71	66	GUC	V	474	439
GAU	D	33	0	GGU	G	11	0	GCU	A	543	0	GUU	V	126	0
CAA	Q	215	209	CGA	R	4	1	CCA	P	131	123	CUA	L	33	28
CAG	Q	11	8	CGG	R	54	52	CCG	P	81	68	CUG	L	151	121
CAC	H	452	424	CGC	R	367	5	CCC	P	121	106	CUC	L	1624	1230
CAU	H	23	0	CGU	R	85	83	CCU	P	504	17	CUU	L	228	44
UAA	*	0	0	UGA	*	9	0	UCA	S	69	53	UUA	L	26	23
UAG	*	0	0	UGG	W	2344	2140	UCG	S	36	11	UUG	L	34	33
UAC	Y	1449	1332	UGC	C	878	837	UCC	S	806	735	UUC	F	1465	1323
UAU	Y	11	0	UGU	C	1	0	UCU	S	198	0	UUU	F	20	0

C) Database composition by 3′-base of predicted specifier sequence.

Specifier	# Sequences	# Sequences (w/ tRNA)	%
NNA	2222	1942	87.4%
NNU	4790	4288	8.6%
NNC	13743	11801	85.9%
NNG	2186	189	89.5%

Table S2. Summary of RNA folding energies for sequences in the database. Mean and standard deviation ΔG_{anti}, ΔG_{term}, $\Delta\Delta G_{term\text{-}anti}$ for all sequences in the database, grouped by tRNA family

	ΔG_{anti} (kcal/mol)		ΔG_{term} (kcal/mol)		$\Delta\Delta G_{term\text{-}anti}$ (kcal/mol)	
tRNA family	μ	σ	μ	σ	μ	σ
ALA	-7.1	2.2	-16.2	7.5	-9.1	6.7
ARG	-5.9	2.2	-17.4	5.4	-11.5	4.4
ASN	-7.2	2.2	-20.0	6.3	-12.9	5.6
ASP	-7.5	2.6	-19.8	7.7	-12.6	6.6
CYS	-5.7	1.8	-19.1	5.5	-13.5	5.3
GLN	-8.1	2.8	-21.8	5.8	-13.6	5.0
GLU	-6.9	1.8	-19.5	5.7	-12.8	4.8
GLY	-9.1	2.9	-24.1	12.0	-15.0	10.5
HIS	-6.1	2.4	-20.0	5.2	-13.9	4.6
ILE	-11.1	7.9	-19.2	10.2	-12.2	8.2
LEU	-8.0	3.1	-20.5	7.3	-12.5	6.2
LYS	-7.4	2.2	-21.0	5.9	-13.6	4.8
MET	-5.8	2.8	-20.4	6.9	-14.7	6.8
PHE	-6.7	2.3	-17.4	5.7	-10.7	5.5
PRO	-7.1	2.0	-19.1	6.3	-12.1	5.9
SER	-7.4	2.2	-19.2	6.8	-11.8	6.4
THR	-6.8	2.5	-19.4	5.7	-12.6	5.1
TRP	-6.7	2.5	-19.9	6.4	-13.4	6.1
TYR	-6.6	2.0	-18.5	5.2	-12.0	5.0
VAL	-5.5	2.3	-18.6	8.2	-13.1	6.9

Fig S1. Consensus sequence and structure for T-box riboswitch and tRNA families in the TBDB. A) T-box riboswitch consensus sequence from the TBDB with important structural features highlighted. Specifier sequence is highlighted in blue, while the T-box riboswitch sequence is highlighted in green. **B**) Consensus tRNA sequence for tRNAs that match with T-box riboswitches in the TBDB. Anticodon is highlighted in blue, while the acceptor arm is highlighted in green. Sequences and structures were generated using cmalign, and trimmed using trimal [16]. 'N' bases are shown where no base was present in >50% of sequences. **C**) Three specifier frames within the specifier bulge are considered in model decision.

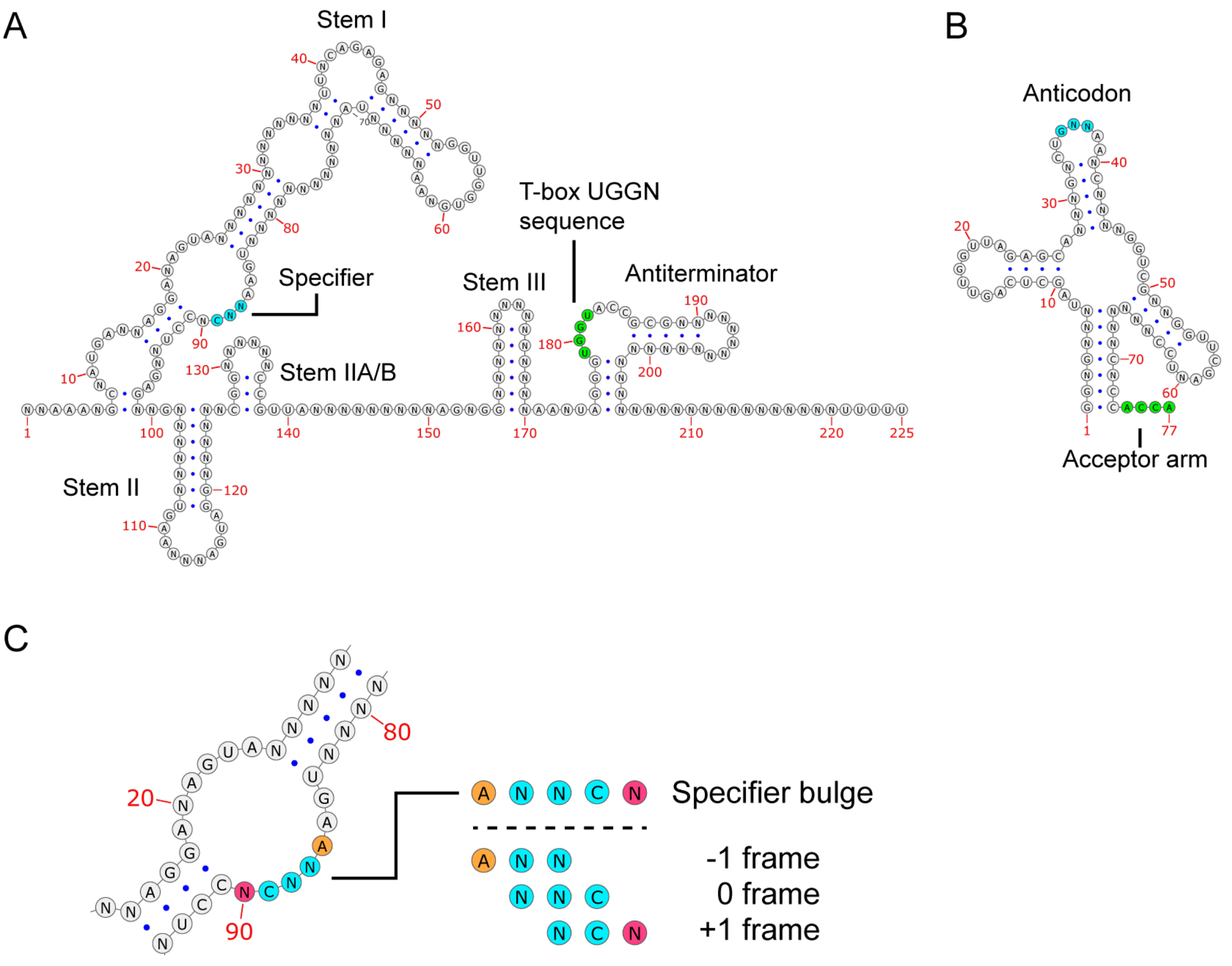

Fig S2. R2R representation of Class II T-box riboswitch covariance model. All Class II T-box riboswitches from the TBDB were aligned using cmalign, and R2R was used to generate a representation based on the covariance between positions [9]. Positions with significant covariation are highlighted in green, absolutely conserved positions are highlighted in red, and positions where one nucleotide (a G or U) is conserved and the other varies are highlighted in blue.

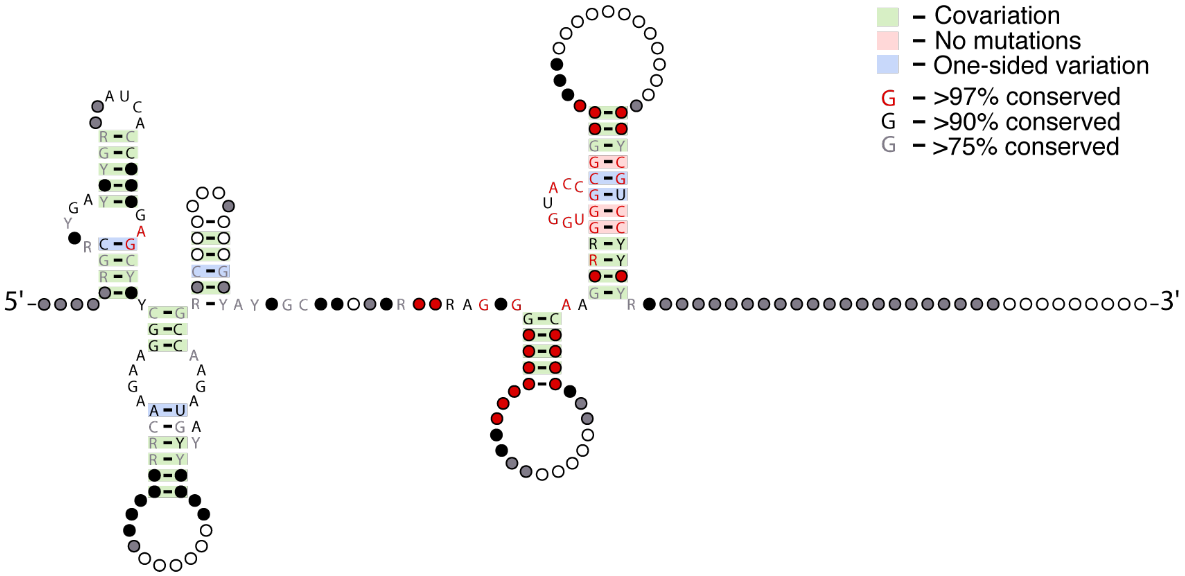

Fig S3. Stem I lengths in TBDB. Stem I length distribution (in nt) of T-box riboswitches in the TBDB, by covariance model used for structure prediction on a A) linear and B) log scale.

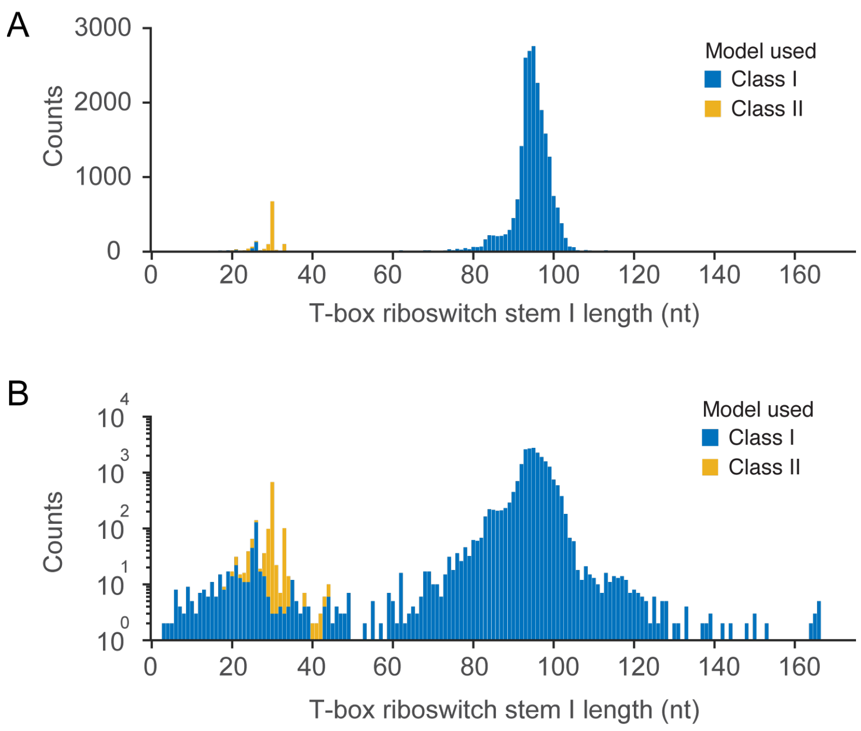

Fig S4. INFERNAL score density plot for sequences in the TBDB. The INFERNAL score is a measure of how well the sequence matches the covariance model, and corresponds to the log-odds ratio of the hit sequence relative to random sequence. Sequences scoring <15 (equivalent to an E-value of >0.05) were removed from the database.

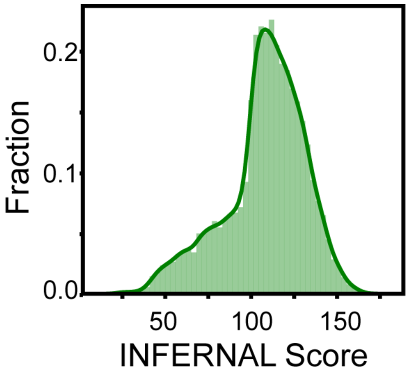

Fig S5. Distribution of minimum free energy structures in the TBDB. A) Distribution of sequences by Gibbs free energy of folding for antiterminator structure (ΔG_{anti} , kcal/mol). **B**) Distribution of sequences by Gibbs free energy of folding for terminator structure (ΔG_{term} , kcal/mol). **C**) Distribution of sequences by change in Gibbs free energy between terminator and antiterminator conformation ($\Delta\Delta G_{term\text{-}anti}$, kcal/mol). **D**) Density plots showing distributions for change in Gibbs Free Energy ($\Delta\Delta G_{term\text{-}anti}$) for T-box riboswitches, sorted by tRNA family.

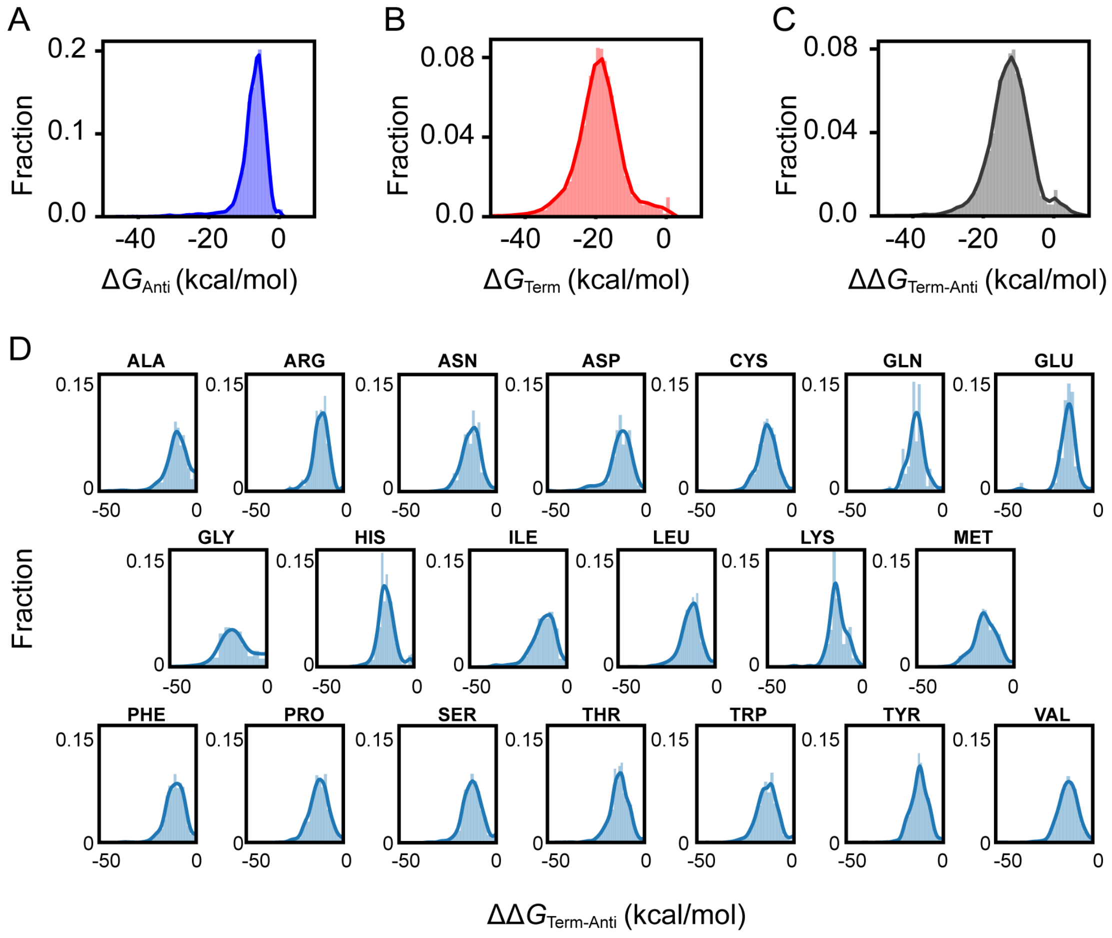

Fig S6. The TBDB interface – Database table. A) Entries into the TBDB are searchable by a variety of fields including sequence, specifier, tRNA family, amino acid family, discriminator bases, and downstream protein. **B**) The 'TBID' column displays name of T-box riboswitch by TBDB unique ID. Clicking on an individual ID brings users to a T-box riboswitch entry page, where additional information is displayed. **C**) The 'Host organism' column displays species from which the T-box riboswitch input sequence was obtained from. **D**) Clicking on genomic accession in the 'Accession' column brings users to an NCBI genetic locus entry page for a particular T-box riboswitch. **E**) The 'Specifier' column shows predicted specifier sequence (5′ to 3′) for given T-box riboswitch. F) The 'UGGN' column shows the T-box bulge 5'-UGGN-3' sequence, which is complementary to the tRNA acceptor end. **G**) 'tRNA' column indicates the type of tRNA, alongside the corresponding amino acid, the T-box riboswitch specifier likely binds. **H**) A description of the protein found downstream of the T-box riboswitch is provided in the 'Downstream protein' column.

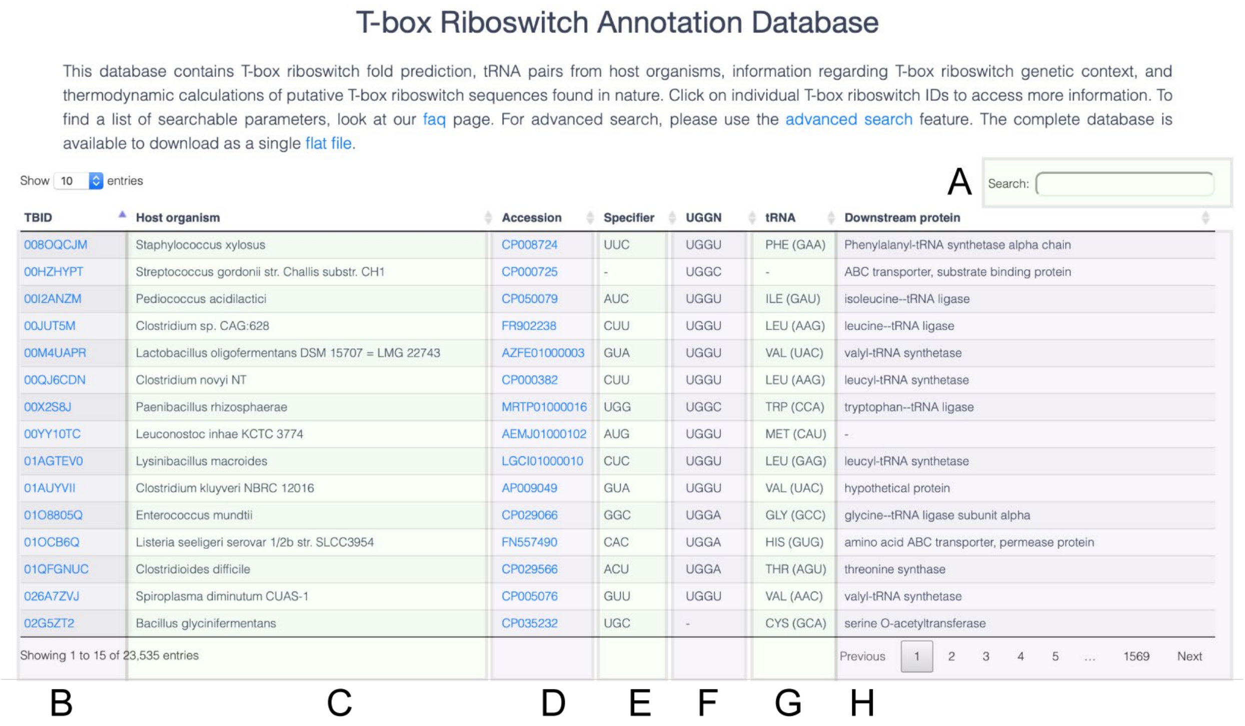

TBID	Host organism	Accession	Specifier	UGGN	tRNA	Downstream protein
008OQCJM	Staphylococcus xylosus	CP008724	UUC	UGGU	PHE (GAA)	Phenylalanyl-tRNA synthetase alpha chain
00HZHYPT	Streptococcus gordonii str. Challis substr. CH1	CP000725	-	UGGC	-	ABC transporter, substrate binding protein
00I2ANZM	Pediococcus acidilactici	CP050079	AUC	UGGU	ILE (GAU)	isoleucine--tRNA ligase
00JUT5M	Clostridium sp. CAG:628	FR902238	CUU	UGGU	LEU (AAG)	leucine--tRNA ligase
00M4UAPR	Lactobacillus oligofermentans DSM 15707 = LMG 22743	AZFE01000003	GUA	UGGU	VAL (UAC)	valyl-tRNA synthetase
00QJ6CDN	Clostridium novyi NT	CP000382	CUU	UGGU	LEU (AAG)	leucyl-tRNA synthetase
00X2S8J	Paenibacillus rhizosphaerae	MRTP01000016	UGG	UGGC	TRP (CCA)	tryptophan--tRNA ligase
00YY10TC	Leuconostoc inhae KCTC 3774	AEMJ01000102	AUG	UGGU	MET (CAU)	-
01AGTEV0	Lysinibacillus macroides	LGCI01000010	CUC	UGGU	LEU (GAG)	leucyl-tRNA synthetase
01AUYVII	Clostridium kluyveri NBRC 12016	AP009049	GUA	UGGU	VAL (UAC)	hypothetical protein
01O8805Q	Enterococcus mundtii	CP029066	GGC	UGGA	GLY (GCC)	glycine--tRNA ligase subunit alpha
01OCB6Q	Listeria seeligeri serovar 1/2b str. SLCC3954	FN557490	CAC	UGGA	HIS (GUG)	amino acid ABC transporter, permease protein
01QFGNUC	Clostridioides difficile	CP029566	ACU	UGGA	THR (AGU)	threonine synthase
026A7ZVJ	Spiroplasma diminutum CUAS-1	CP005076	GUU	UGGU	VAL (AAC)	valyl-tRNA synthetase
02G5ZT2	Bacillus glycinifermentans	CP035232	UGC	-	CYS (GCA)	serine O-acetyltransferase

Fig S7. The TBDB interface – Entry title and source information. A) Entries into the TBDB are titled using the TBDB unique ID. **B**) A brief description of the T-box riboswitch is also provided, and includes tRNA family displayed as amino acid (anti-codon), and host organism where T-box riboswitch input sequence was found. **C**) The 'Source Information' panel provides a summary of important T-box riboswitch features.

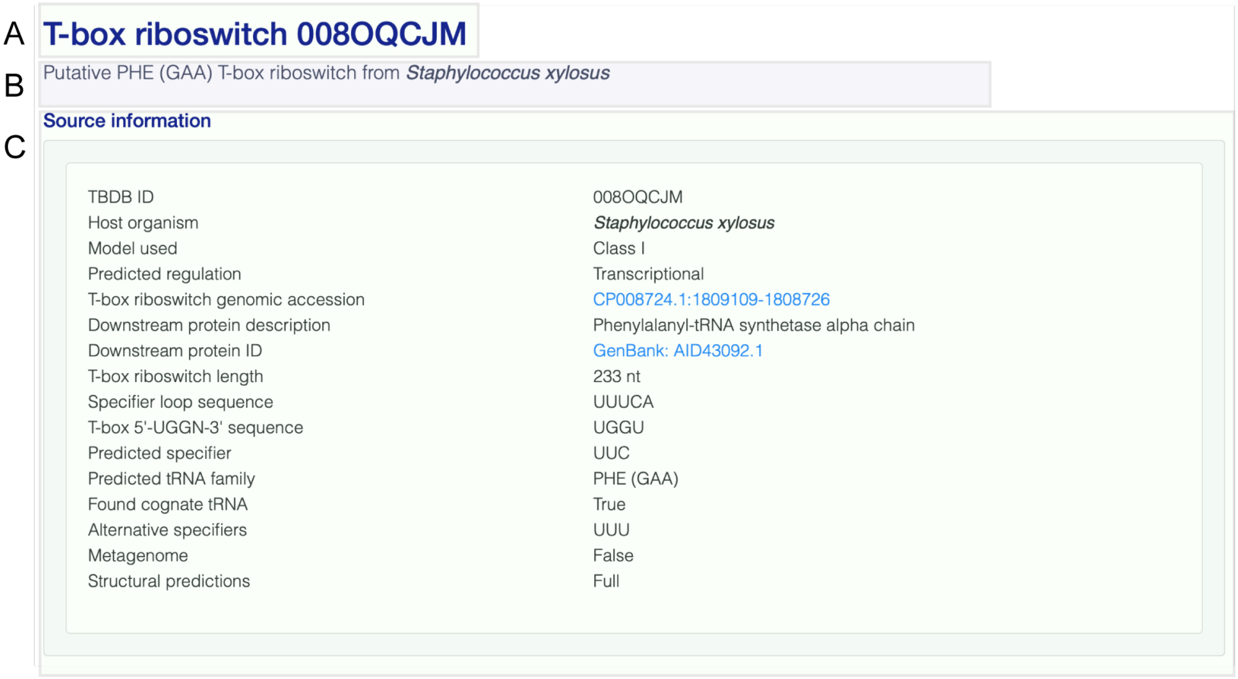

Fig S8. The TBDB interface – Genomic context viewer. An integrated NCBI graphical display of the T-box riboswitch genomic region can be used to examine genes or operons controlled by the riboswitch. Here, a Trp T-box riboswitch is shown upstream of a tryptophan biosynthesis operon. **A**) The browsing menu allows users to change a variety of settings for the NCBI graphical viewer. **B**) The T-box riboswitch input sequence is highlighted in blue, with arrows indicating the direction, (+) or (-) strand of T-box riboswitch transcription. **C**) The genomic region is shown as the top gray bar, with numbers indicating genomic locus. **D**) Genes are provided in the center panel and are annotated as provided by NCBI.

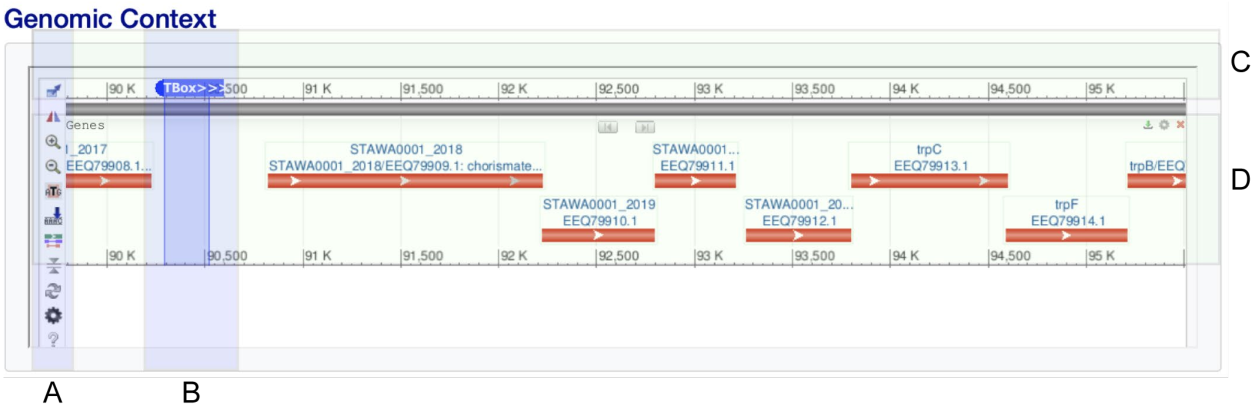

Fig S9. The TBDB interface – T-box riboswitch FASTA sequence. The FASTA sequence of the T-box riboswitch, starting from Stem I and ending at the terminator, is provided as a panel. Sequence header uses the TBDB unique ID.

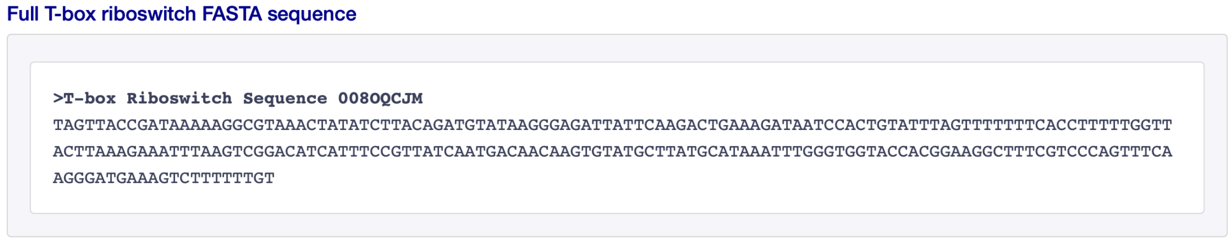

Fig S10. The TBDB interface – Predicted secondary structure visualization. Secondary structures for T-box riboswitches, as predicted by folding and refinement, are provided as dot-bracket structures for antiterminator and terminator. The dot-bracket representations can be used alongside the FASTA sequence provided in the "Full T-box riboswitch FASTA sequence" panel to generate custom 2-D representations of T-box riboswitches. Headers use TBDB unique ID for each T-box riboswitch. **A**) Two secondary structure representations are provided, if available, for each T-box riboswitch sequence. For putative transcriptional T-box riboswitches, the 'Antiterminator' option shows the antiterminator fold while the 'Terminator' option shows the terminator fold representation. Sequences are displayed using VARNA flat representation for **B**) antiterminator and **C**) terminator conformations. Gaps are inserted after the Stem I structure to improve clarity of display and prevent overlaps between the stem-loops in visualization. Important features are highlighted as follows: Stem I (light yellow), specifier bases (orange), antiterminator (light blue), anti-acceptor arm (blue), and terminator (red). Note that terminator representations are not displayed for sequences where local fold using ViennaRNA failed to find a MFE terminator structure.

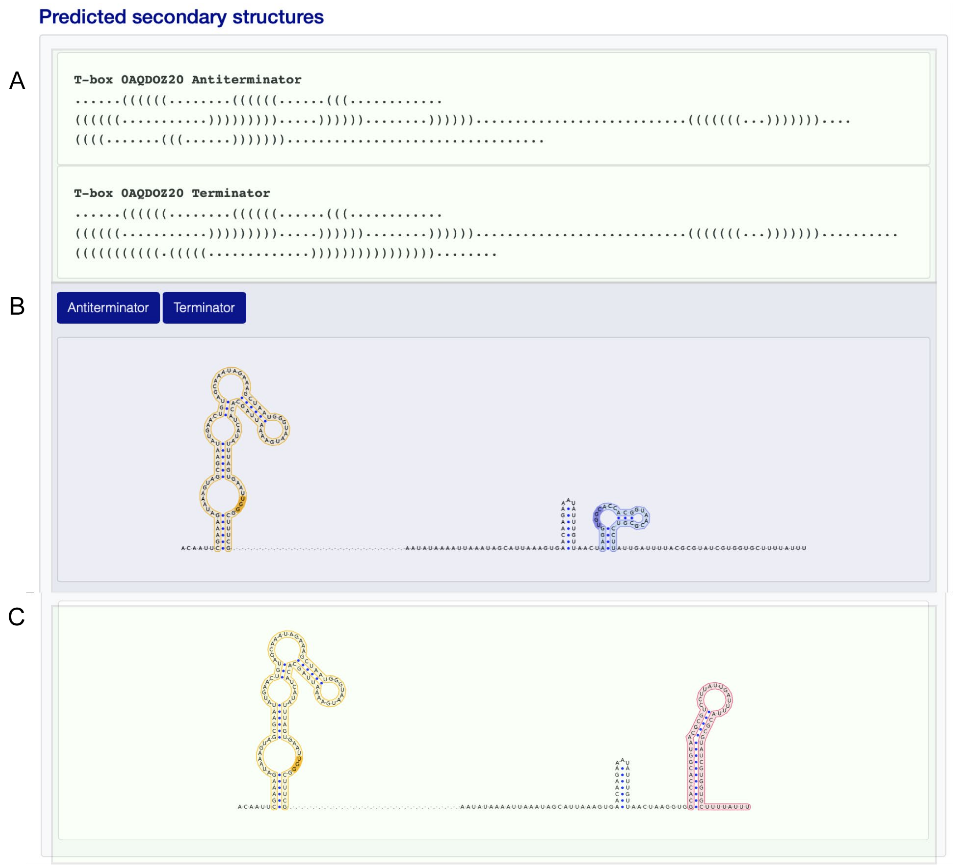

Fig S11. The TBDB interface – tRNA scan results. Results from tRNA scan for a matching tRNA from host organism are provided in the 'tRNA prediction' panel. tRNAscan-SE was used on the host organism to identify tRNA with anticodons matching the specifier sequence of the given T-box riboswitch. **A**) The sequence and structure, in dot-bracket notation, for the top tRNA match is shown. FASTA header displays tRNA family and host organism. If no tRNA was found in the host, a null result will be displayed. If there was ambiguity in deciding the specifier frame, results for alternative specifier frames are also displayed. **B**) For T-box riboswitches where a matching tRNA from native host was found, secondary structure is displayed as a 2-D VARNA generated image.

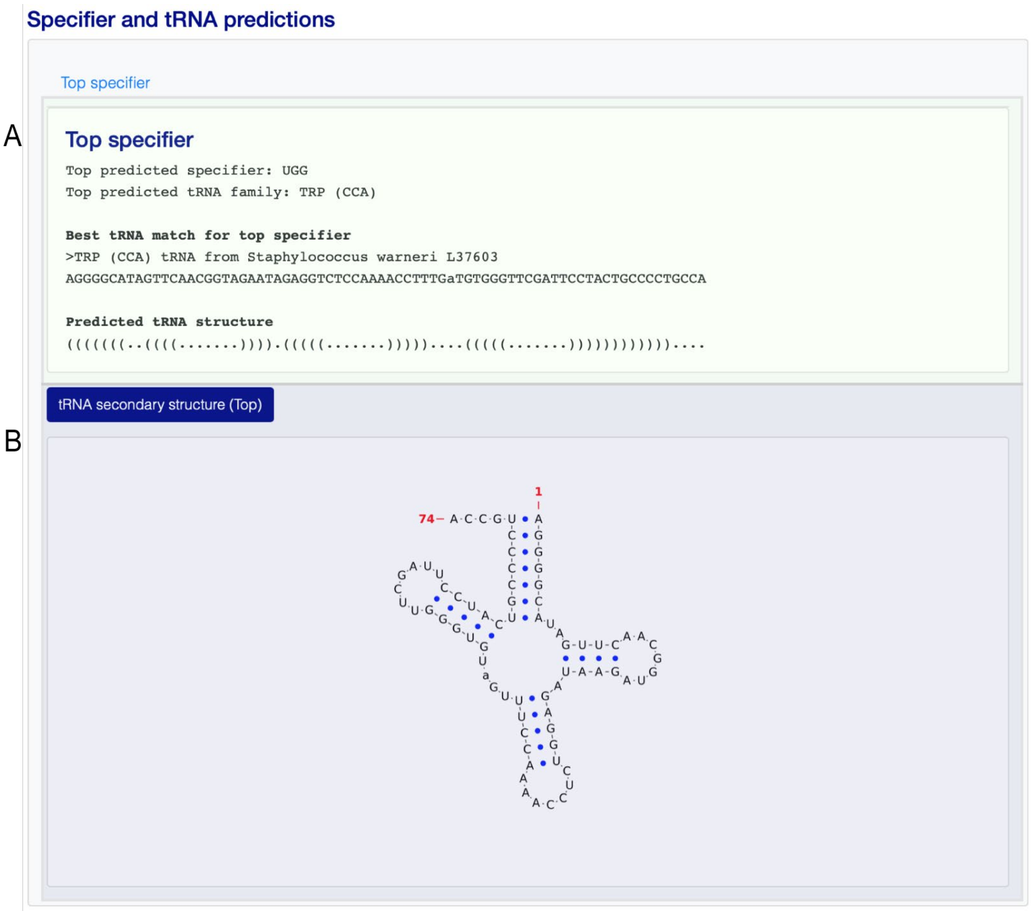

Fig S12. The TBDB interface – MFE predictions. Gibbs free energies from MFE predictions (Vienna RNA) are provided for **A**) antiterminator structure, **B**) terminator structure. **C**) Displays the difference in MFE between terminator and antiterminator conformations. For T-box riboswitches where folding of terminator/sequestrator or antiterminator/antisequestrator failed, the corresponding MFE is not displayed.

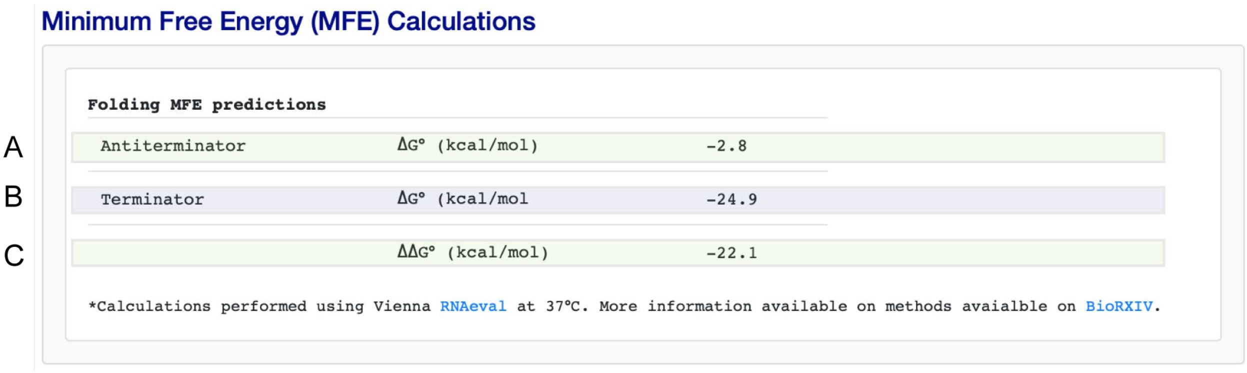

Fig S13. The TBDB interface – INFERNAL prediction output. Information used to predict T-box riboswitch structures using INFERNAL is provided in the 'Infernal T-box riboswitch prediction output' panel [7]. **A**) INFERNAL quality score metrics (E-Value, score, bias, and covariance model accuracy (CM)) are provided. **B**) Feature identification output, using input sequence of T-box, gives the position information for Stem I, antiterminator, terminator, and specifier. All stems found using the INFERNAL model are provided as 'All Stems'. Lengths of each structure are displayed. Loci are shown in [index start, index end] format. **C**) Input sequence (as obtained from source database) that was used for T-box riboswitch predictions is shown in FASTA format. The header indicates host organism and genomic accession with locus start-end. **D**) INFERNAL output sequence is provided in FASTA format, as well as E) INFERNAL output structure.

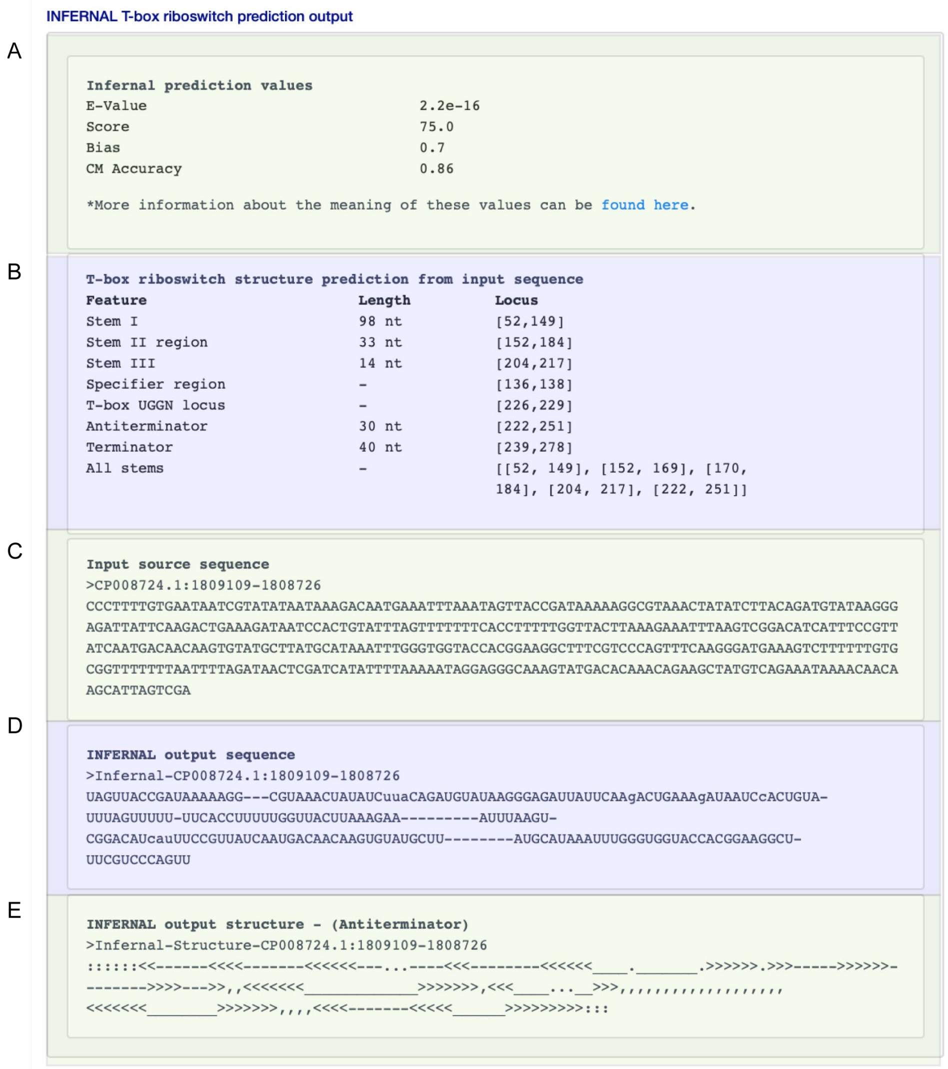

Fig S14. Composition of TBDB by phyla and T-box riboswitch family. TBDB contains 23,535 T-box riboswitch sequences from 3,632 different species. The majority of sequences (20,683) in our collection originate from the phyla Firmicutes. T-box riboswitches shown are displayed in decreasing order of abundance in the TBDB collection, with Ile being the most commonly represented and Glu the least. Phyla were clustered by hierarchical clustering using binary distance metric from the Superheat R package [17]. Heatmap displays abundance (total number) of individual T-box riboswitch families for each phyla in our TBDB collection, using a $\log_{10}$ scale to visualize low abundance distribution.

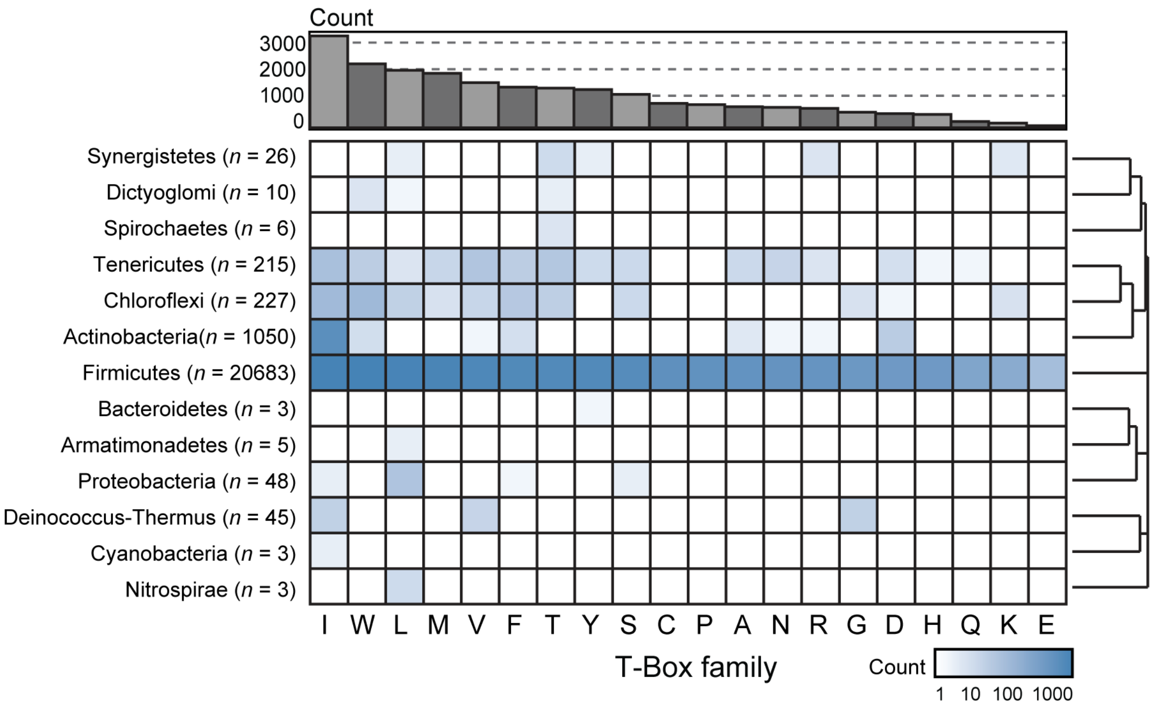

Supplement: gkaa721_Supplemental_Files [file gkaa721_supplemental_files.zip › TBDB_NAR_SI.pdf]
